# Soybean aphid, *Aphis glycines* (Hemiptera: Aphididae), developmental and reproductive capacity on white clover, *Trifolium repens* (Rosales: Leguminosae), in northeast China

**DOI:** 10.1007/s13355-017-0500-5

**Published:** 2017-06-05

**Authors:** Xiaohui Chen, Yanjie Fan, Wei Zhang, Zhenqi Tian, Jian Liu, Kuijun Zhao

**Affiliations:** 1 Department of Plant Protection, College of Agriculture, Northeast Agricultural University, Harbin, 150030 China; 2 Department of Statistics, College of Mathematical Science, Harbin Normal University, Harbin, 150025 China

**Keywords:** Soybean aphid, White clover, Clip cage

## Abstract

Nymphs of *Aphis glycines* Matsumura were individually reared to adults in the laboratory on detached leaf discs of *Trifolium repens* L. (white clover) mounted on agar medium. Adults of *A. glycines* were fed *T. repens* within small clip cages in the field. Development, reproduction and intrinsic rates of increase of *A. glycines* were studied. These data were compared to those of controls fed known host plants including cultivated soybean *Glycine max* (L.) Merr. and the wild soybean species *Glycine soja* Sieb & Zucc. The results demonstrated that nymphs of *A. glycines* successfully developed into adults and reproduced efficiently when reared on *T. repens* in the laboratory. The lower development temperature threshold for nymphs fed *T. repens* was estimated as 8.27 °C, and the effective cumulative temperature for *A. glycines* development from nymph to adult was 90.91 degree-days. Adults of *A. glycines* could also survive on *T. repens* in the field, but only a few nymphs were produced.

## Introduction

The soybean aphid, *Aphis glycines* Matsumura, is an insect pest commonly found on soybeans in Asia. In 2000, *A. glycines* became established in North America (Ragsdale et al. 2004, 2011). By 2003, this pest had infested over 21 America states and three Canadian provinces (Venette and Ragsdale 2004). Soybean aphids can cause direct damage to plants by sucking fluids from leaves and stems (Liu and Zhao 2007; Wu et al. 2004). Additionally, they are also capable of transmitting a variety of plant viruses (Davis et al. 2005; Mueller and Grau 2007).

Secondary hosts of *A. glycines* include cultivated soybean *Glycine max* (L.) Merr., and the wild soybean species *Glycine soja* Sieb & Zucc (Wang et al. 1962). It was determined that the aphid could likely utilize horsenettle, *Solanum carolinense* L. (Clark et al. 2006), and Japanese *Metaplexis*, *Metaplexis japonica* (Thunb.) Makino (Chen et al. 2015) as hosts. Results from a previous study showed that *Trifolium repens* L. was a poor host for soybean aphid (Hill et al. 2004). A later study showed that clover variety significantly affected aphid density, and *A. glycines* could achieve highest population growth on Ladino, a variety of white clover (Swenson et al. 2014).

*T. repens* is a common legume in natural landscapes and cultivated fields and has a wide distribution in northeast China. In this latter region, it is still questionable whether *A. glycines* can utilize *T. repens* as a host. Understanding the role of this widely distributed plant as a host for *A. glycines* is important for the effective management of this insect in northeast China.

In the current study, nymphs of *A. glycines* were individually reared to adults on detached leaves of *T. repens* in the laboratory. Adults of *A. glycines* were fed *T. repens* in the field while contained in small clip cages. Development, reproduction, and intrinsic rates of increase of *A. glycines* were studied. These data were compared to those of controls fed the species’ known hosts *G. max* and *G. soja*.

## Materials and methods

### Aphid source and host plants

Soybean aphids were taken from a soybean field at the Xiangfang Experiment Station, Northeast Agricultural University (NEAU), Harbin, Heilongjiang Province, northeast China (126.75°E, 45.72°N). The colony was maintained on *G. max* (variety Heinong 51) in an environmental chamber at 25 ± 1 °C, 70 ± 5% relative humidity (RH), and a photoperiod of 14:10 h (light:dark; L:D) with artificial light of 12,000 lx. *G. max* was grown in a chamber at 28 ± 1 °C with six to ten seeds per pot in 10 × 10-cm (diameter × height) plastic pots under the same humidity and photoperiod as described above. Seedlings 15–20 cm tall at the V2 growth stage (Fehr and Caviness 1977) were used for experiments. *T. repens* (variety Rivendel), were collected from a lawn in NEAU (126.75°E, 45.72°N) and were transplanted into a 50-m^2^ experiment plot. These plants were used for experiments when they were at a vegetative or generative growth phase. Seeds of *G. soja* were collected from a field near Limin, Harbin (126.61°E, 45.87°N) and were planted in the same plot. These plants were allowed 4–5 weeks to germinate and mature before being used for experiments.

### Development of nymphs

**Fig. 1:**
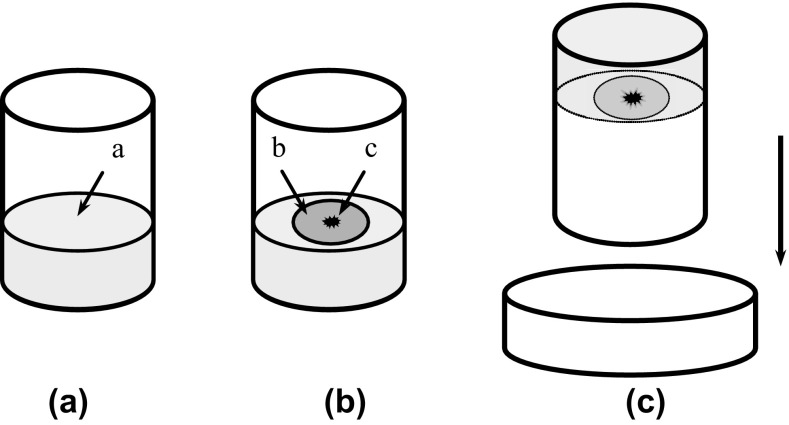
Method for breeding individual *Aphis glycines.* **a** A 50-mL glass beaker with 10 mL solid agar medium (*a*), **b** *A. glycines* nymph (*c*) placed on the reverse side of a leaf disc (*b*) adhered to the surface of the solid agar medium, **c** the glass beaker placed upside down on a 5-cm diameter Petri dish

About 200 apterous adult aphids were transferred from the stock colony onto ten pots of soybean plants (approximately 20 aphids per pot). The plants were placed in an environmental chamber at 25 ± 1 °C, 70 ± 5% RH and a 14:10-h (L:D) photoperiod for a 24-h reproduction period, after which, all adults were removed. Using a small brush, newly deposited nymphs were individually removed from the plants. Detached leaves of *G. max*, *G. soja*, and *T. repens* were cut into 1.5 cm-diameter leaf discs using a hole punch. Solid agar media were prepared in 45-mL, 4 × 4.5-cm (diameter × height) glass beakers. Each nymph was placed on the reverse side of a leaf disc adhered to the surface of the medium. The beaker was then placed upside down on a 5-cm-diameter Petri dish (leaf disc method) (Fig. 1) (Liu 1987; Liu et al. 2003). For each host plant treatment, 50 nymphs were tested at each temperature. Beakers were placed in environmental chambers at 13, 18, 23, 28, and 33 ± 1 °C, 70 ± 5% RH, and a 14:10-h (L:D) photoperiod. Individual aphids were checked daily for ecdysis and survivorship. Leaves and media were replaced every 5–7 days when the old leaves became yellowish or upon observation of fungal growth.

### Adult longevity and reproductive capacity

Adults which had been reared from nymphs at 13–33 ± 1 °C were maintained in the same conditions as the immature insects. Nymphs deposited by each female were counted and removed daily. Adult longevity was recorded daily until the death of each adult. Leaves and media were replaced every 5–7 days when leaves became yellowish or upon observation of fungal growth.

### Adult development and fecundity in the field

**Fig. 2:**
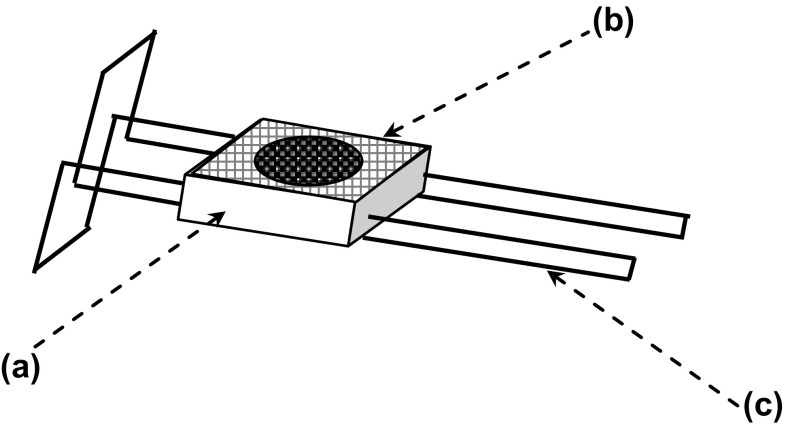
Clip cage used for field plants. Piece of KT board (polystyrene) with a circular 1.0-cm hole (*a*), **b** polyester net with 1 × 1-mm mesh (*b*), **c** T-form frame constructed from elastic steel wire (*c*)

A small clip cage was designed for this experiment. Pieces of polystyrene KT board, 2.0 cm × 2.0 cm × 0.5 cm (length × width × height), were used for the clip cages. Firstly, a circular 1.0-cm hole was cut in the middle of this piece with a hole punch. Then the hole was covered with polyester net with 502-glue (Tonglin Glue Manufactory, Harbin, China). Finally, a T-form frame consisting of elastic steel wire was set into the piece (Fig. 2).

Another 100 *A. glycines* nymphs were fed soybean using the leaf disc method at 23 ± 1 °C, 70 ± 5% RH and a 14:10-h (L:D) photoperiod. When they developed into adults, 27, 32, and 31 soybean aphids were respectively transferred onto leaves of *G. max*, *G. soja*, and *T. repens* growing in a 10-m^2^ plot. Each aphid was covered by a clip cage and surveys were conducted from 24 August to 19 September 2012. Nymphs deposited by each adult were counted and removed daily with a small brush. Adult longevity was recorded daily until the death of each adult.

### Data analysis

Raw data for all individuals reared at 13–33 ± 1 °C were analyzed according to the age-stage, two-sex life table theory (Chi 1988). Nymph stage, adult longevity, adult fecundity were calculated using the bootstrap method (Efron and Tibshirani 1993) in TWOSEX-MSChart software (Chi 2015). Differences in these parameters among hosts at each temperature were analyzed by general linear model (GLM) and Tukey’s honest significant difference (HSD) tests. Differences in longevities and fecundities of adults among field-grown hosts were also analyzed by GLM and HSD tests. Analyses were conducted with SAS 8.1 software (SAS Institute 2000).

Intrinsic rates of increase for *A. glycines* on different hosts at each temperature were calculated using the bootstrap technique (Efron and Tibshirani 1993) in the TWOSEX-MSChart program (Chi 2015). Because bootstrap analysis uses random resampling, a small number of replications will generate variable means and SEs. To reduce the variability of the results, we used 100,000 bootstrap iterations. We then used the paired bootstrap test to examine the differences among the three hosts at each temperature (Efron and Tibshirani 1993).

To estimate the lower developmental temperature threshold and effective cumulative temperature for nymph development on each plant, linear regression of the mean developmental rate *y* (the reciprocal of development time to adult) on temperature *x* was applied to each temperature from 13 to 28 °C (Murai 2000), and was performed with the GLM.

## Results

### Development of nymphs

**Fig. 3:**
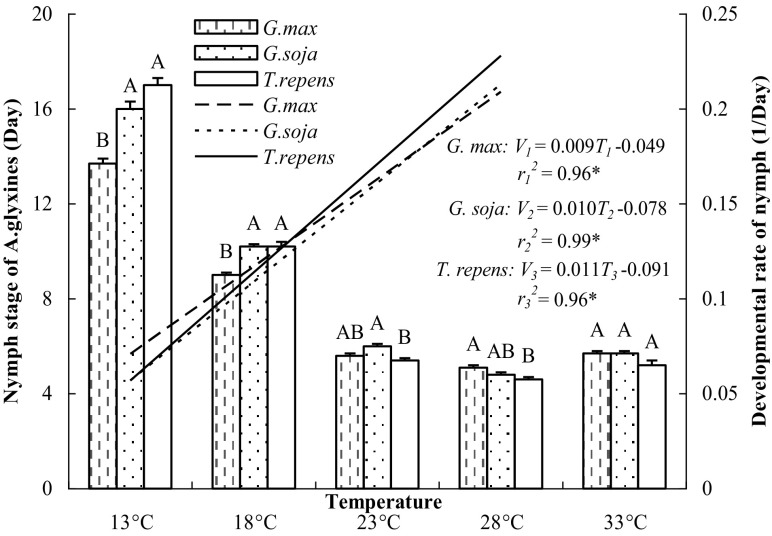
Nymph stage of *A. glycines* feeding on three species of plants at five constant temperatures. Nymphs of *A. glycines* on different plant species at each temperature followed by the* same letter* do not differ significantly [*p* < 0.01; honest significant difference (HSD) test]. Linear regression of mean developmental rate of *A. glycines* nymphs on different plant species (*y*) on temperature (x) was applied to each temperature (13–28 °C). The data for 33 °C were omitted because the adverse effects of high temperature were evident (Tables 1, 2). The three regression lines and their equations are also shown. *Asterisk* denotes a significant difference at *p* < 0.05. *V*_1_, *V*_2_, *V*_3_ Developmental rate of *A. glycines* nymphs on different plant species; *T*_1_, *T*_2_, *T*_3_ average daily temperature during nymph stage

Nymphs of *A. glycines* successfully developed into adults when they were fed *G. max, G. soja*, and *T. repens* at temperatures of 13–33 °C. Significant linear relationships between mean developmental rate and temperature (between 13 and 28 °C, inclusive) were evident for nymphs feeding on different plants (Fig. 3).

When these linear regression equations (Fig. 3) for nymphs fed *G. max, G. soja* and *T. repens* were deformed as 1/*V*_*1*_ = 111.11/(*T*_*1*_−5.44), 1/*V*_*2*_ = 100.00/(*T*_*2*_−7.80) and 1/*V*_*3*_ = 90.91/(*T*_*3*_−8.27), the respective lower development temperature thresholds for nymphs were estimated as 5.44, 7.80 and 8.27 °C; the effective cumulative temperatures for *A. glycines* development from nymph to adult were estimated as 111.11, 100.00 and 90.91 degree-days, respectively.

### Adult longevity, reproductive capacity, and intrinsic rates of increase

Adult longevities, fecundities, and intrinsic rates of increase of *A. glycines* fed the three plant species at five constant temperatures are shown in Table 1.

**Table 1:** Longevities, fecundities, and intrinsic rate of increase (*r*_*m*_) for *A. glycines* adult feeding on three plant species at five constant temperatures

Temperature (°C)	Host plants	Adult no.	Longevity of adult (days) (mean ± SE)^a^	No. of progeny/adult (mean ± SE)^a^	*r*_*m*_ (mean ± SE)^b^
13	*Glycine max*	50	32.5 ± 2.6 AB	28.2 ± 1.5 A	0.2 ± 2.4E−003 a
	*Glycine soja*	42	22.1 ± 1.6 B	22.8 ± 1.6 AB	0.1 ± 4.2E−003 b
	*Trifolium repens*	32	37.4 ± 3.0 A	17.3 ± 1.4 B	0.1 ± 5.6E−003 c
18	*G. max*	50	18.4 ± 1.0 A	40.8 ± 1.6 B	0.3 ± 3.9E−003 a
	*G. soja*	48	17.6 ± 0.9 A	52.1 ± 1.7 A	0.2 ± 3.6E−003 b
	*T. repens*	43	14.6 ± 1.3 A	25.3 ± 2.1C	0.2 ± 7.2E−003 c
23	*G. max*	48	15.1 ± 1.1 AB	46.1 ± 2.1 A	0.4 ± 6.2E−003 ac
	*G. soja*	48	12.6 ± 0.9 B	29.6 ± 1.5 B	0.4 ± 8.9E−003 b
	*T. repens*	46	17.3 ± 0.9 A	51.1 ± 1.8 A	0.4 ± 7.9E−003 c
28	*G. max*	44	12.0 ± 0.6 A	29.8 ± 1.9 B	0.4 ± 10.6E−003 b
	*G. soja*	49	9.1 ± 0.5 B	40.1 ± 1.9 A	0.5 ± 7.6E−003 a
	*T. repens*	39	7.3 ± 0.7 B	23.9 ± 2.1 B	0.4 ± 15.6E−003 b
33	*G. max*	41	3.2 ± 0.2 A	–	–
	*G. soja*	36	2.7 ± 0.3 AB	0.1 ± 0.1	−0.4 ± 89.7E−003
	*T. repens*	13	1.8 ± 0.5 B	–	–

Initial aphid numbers were all 50 when *r*_*m*_ were calculated

^a^Means* at each temperature* followed by the* same letter* do not differ significantly [*p* < 0.01; honest significant difference (HSD) test]

^b^Means* at* *each temperature* followed by the* same letter* do not differ significantly (*p* < 0.05; bootstrap)

### Adult development and fecundity in the field

The data demonstrated that *A. glycines* adults could survive on *T. repens* in the field. Adult longevities on *T. repens* were similar to those on *G. max*, but were shorter than those on *G. soja* (ANOVA; *F* = 6.35; *p* < 0.01). Adults of *A. glycines* fed *T. repens* had lower fecundities than those fed *G. max* and *G. soja* (ANOVA; *F* = 23.58; *p* < 0.01) (Table 2).

**Table 2:** Longevities and fecundities of *A. glycines* adults feeding on three plant species in the field

Host plants	Adult no.	Longevity of adult (days) (mean ± SE)	No. of progeny/adult (mean ± SE)
*G. max*	27	8.7 ± 3.6 AB	15.0 ± 5.8 A
*G. soja*	32	10.0 ± 5.5 A	12.8 ± 7.0 A
*T. repens*	31	4.2 ± 0.8 B	0.1 ± 0.3 B

Means followed by the* same letter* do not differ significantly (*p* < 0.01; HSD test)

## Discussion

There has been controversy as to whether *A. glycines* could utilize *T. repens* as a host. Results from a previous study showed that *T. repens* was a poor host for soybean aphids (Hill et al. 2004). High levels of *A. glycines* population growth were observed on the Ladino variety of white clover (Swenson et al. 2014). Morphological variations of *A. glycines* were studied when they were fed *T. repens* (Chen et al. 2015). This study was conducted to determine the developmental and reproductive capacities of *A. glycines* on *T. repens*, and our results indicated that *A. glycines* could feed on *T. repens* in northeast China (Chen et al. 2015).

In our study, the lower development temperature threshold for nymphs on soybean was estimated at 5.44 °C, which differs from the previous estimates of 1.66 °C (Xu et al. 2011) and 9.50 °C (Hirano et al. 1996). The effective cumulative temperature for *A. glycines* development from nymph to adult was estimated at 90.91 degree-days, which also differs from the previous estimates of 57.10 degree-days (Hirano et al. 1996) and 202.79 degree-days (Xu et al. 2011).

Interestingly, there were differences in fecundities of *A. glycines* when fed *G. max*, *G. soja*, and *T. repens.* The fecundities also differed when they were reared between 13 and 28 °C. Fecundities of *A. glycines* fed *T. repens* were lower than those fed *G. max* at 13 and 18 °C; nevertheless, they were similar to those fed *G. max* at 23 and 28 °C (Table 1).

We undertook this study to compare the adaptabilities of *A. glycines* to different plants. Although *A. glycines* could survive (Fig. 3) and reproduce well (Table 1) on *T. repens*, experiments were only conducted at constant temperatures. Importantly, the aphids that we utilized were from field populations and there were likely differences among the adaptations of individuals for different hosts. To ascertain more significant experimental conclusions, it would be necessary to conduct experiments with aphids derived from a monoclonal population. In addition, soybean aphids used in this study were apterous ones. Only alate aphids migrate between primary and secondary hosts in the autumn or spring (Liu and Zhao 2007; Wu et al. 2004). With data derived from a study on alate *A. glycines* feeding on *T. repens*, further research is required to address whether *A. glycines* could feed on *T. repens* in northeast China.
